# Comparison between Polybutylcyanoacrylate Nanoparticles with Either Surface-Adsorbed or Encapsulated Brain-Derived Neurotrophic Factor on the Neural Differentiation of iPSCs

**DOI:** 10.3390/ijms20010182

**Published:** 2019-01-06

**Authors:** Martin Hsiu-Chu Lin, Chiu-Yen Chung, Kuo-Tai Chen, Jih-Chao Yeh, Tsong-Hai Lee, Ming-Hsueh Lee, I-Neng Lee, Wei-Chao Huang, Jen-Tsung Yang

**Affiliations:** 1 Department of Neurosurgery, Chang Gung Memorial Hospital, Chiayi 61363, Taiwan; martinhclin@hotmail.com (M.H.-C.L.); yen5103106@gmail.com (C.-Y.C.); tad9116@cgmh.org.tw (K.-T.C.); ming2072@cgmh.org.tw (M.-H.L.); u9201031@gmail.com (W.-C.H.); 2 Stroke Center and Department of Neurology, Linkou Chang Gung Memorial Hospital, Taoyuan 33305, Taiwan; Jihchao@gmail.com (J.-C.Y.); thlee@cgmh.org.tw (T.-H.L.); 3 Department of Urology at the University of Southern California, Los Angeles, CA 90033, USA; 4 College of Medicine, Chang Gung University, Taoyuan 333, Taiwan; 5 Department of Medical Research, Chang Gung Memorial Hospital, Chiayi 61363, Taiwan; geneasylum@yahoo.com.tw

**Keywords:** brain-derived neurotrophic factor, polybutylcyanoacrylate nanoparticles, superparamagnetic iron oxide, induced pluripotent stem cell

## Abstract

The brain-derived neurotrophic factor (BDNF) is vital in the neural differentiation of neural stem/progenitor cells, and together may have therapeutic potential for neural regeneration. In this study, a multiplexed polybutylcyanoacrylate nanoparticle (PBCA NP) delivery platform was constructed, incorporating either surface-adsorbed or encapsulated BDNF for the induction of neural differentiation in induced pleuripotent stem cells (iPSCs), where tween 80 (T80) and superparamagnetic iron oxide (SPIO) were added for central nervous system (CNS) targeting and magnetic resonance (MR) image tracking, respectively. Both methods by which the BDNF was carried resulted in loading efficiencies greater than 95%. The nanoparticle-mediated delivery of BDNF resulted in neural differentiation of iPSCs detected on immunofluorescence staining as early as 7 days, with enhanced differentiation efficiency by 1.3-fold compared to the control on flow cytometry; the delivery system of surface-adsorbed BDNF gave rise to cells that had the best neural development than the encapsulated formulation. T80-coating disrupted the in vitro blood–brain barrier model with a corresponding 1.5- to two-fold increase in permeability. SPIO-loaded PBCA NPs exhibited a concentration-dependent, rapid decay in signal intensity on the phantom MR experiment. This study demonstrates the versatility of the PBCA NP, and the surface-adsorption of BDNF is the preferred method of delivery for the differentiation of iPSCs.

## 1. Introduction

Healing by regeneration of the damaged tissue requires either the direct repair or replacement of the damaged cells by specialized tissue-specific resident stem/progenitor cells [1]. In the central nervous system (CNS), the regenerative capacity following injury or disease is limited, thus it often translates to suboptimal neurologic recovery and poses a unique set of challenges from a therapeutic point of view. In spite of the fact that many regions in the mammalian CNS are known to harbor neural stem cells [2,3,4,5,6,7], and the role that these resident stem cells play in homeostasis and repair, the imbalance of growth-inhibiting and promoting cues [8,9], the non-permissive microenvironment outside the neurogenic region [10], neuro-inflammation [11], and the age-related signal alteration in the stem cell niche with the associated decline in the stem cell pool, all serve to hinder the regenerative process in the CNS [12,13]. These constraints may be resolved in part by stem/progenitor cell-replacement therapy, which not only replenishes the damaged cells by neurogenesis and gliogenesis, but also exerts a neurotrophic and immune-modulatory effect that complements the endogenous reparative mechanism [14,15,16].

The seemingly limitless supply of induced pluripotent stem cells (iPSCs) that can be generated by the reprogramming of somatic cells to exhibit embryonic stem cell-like self-renewal and pluripotent differentiation capabilities created new opportunities for autogenic cell replacement therapy [17,18,19]; however, safety concerns remain with respect to the risk of tumorigenicity and immunogenicity of the transplanted cells which occur as a result of residual iPSCs and transgene-induced genomic instability [20,21,22]. Though methods have been devised to eliminate residual iPSCs in the iPSC-derived product, and safer transgene-free reprogramming protocols have also been developed, a less time-consuming and labor-intensive differentiation technique is still an important requisite for the clinical application of iPSCs [23,24,25]. The brain-derived neurotrophic factor (BDNF) is a common ingredient for the neural differentiation of iPSCs [26,27], and it additionally is a vital trophic factor in the nervous system that maintains the structure and function of neurons, regulates synaptic transmission, and promotes the axonal regeneration and differentiation of neural precursor cells [28,29,30]; therefore, our goal was to create a BDNF formulation that can be used to simplify and shorten the neural differentiation process of iPSCs for cell therapy, and to simultaneously stimulate the endogenous reparative mechanism by crossing the blood–brain barrier (BBB) in a combined “protein-plus-cell” approach.

The tween 80 (T80)-coated polybutylcyanoacrylate nanoparticle (PBCA NP) is a versatile polymeric delivery system that exhibits CNS-targeting capabilities [31,32], and it has been widely studied for the delivery of various substances, including drugs, proteins, and peptides, and genes that are normally impermeable to the BBB into the CNS [33,34,35,36]. The cargos can either be carried by surface adsorption or encapsulation using the easily scalable polymerization or nano-precipitating methods under ambient conditions with the n-butylcyanoacrylate (BCA) monomer or pre-formed PBCA polymers, respectively [37]. However, the cargo-loading efficiency (LE) and release kinetics differ between surface-adsorbed and encapsulated formulations of PBCA NP, in that surface adsorption is associated with a lower cargo LE, and a rapid initial burst-release, followed by a shorter sustained-release profile, whereas nanoparticle disintegration by surface erosion and drug diffusion through the polymeric matrix of the entrapped formulation results in a longer release curve [38]; these key differences may have an impact on the effectiveness of the delivered cargo.

**Scheme 1:**
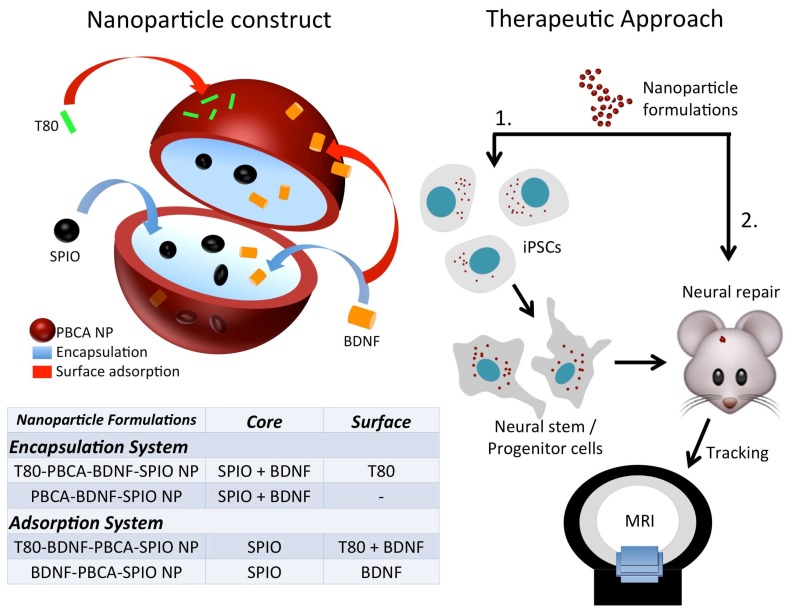
The multiplexed polybutylcyanoacrylate nanoparticle (PBCA NP) delivery platform consists of several components; the brain-derived neurotrophic factor (BDNF) is the inductive agent for neural differentiation of induced pleuripotent stem cells (iPSCs), tween 80 (T80) is the surfactant necessary for enhanced penetrability through to the blood–brain barrier (BBB) into the central nervous system (CNS), and superparamagnetic iron oxide (SPIO) is the tracking agent for magnetic resonance (MR) imaging. The blue and red arrows represent loading of substances by encapsulation and surface absorption respectively. The encapsulation system of BDNF with and without T80 coating is denoted by T80-PBCA-BDNF-SPIO NP, and PBCA-BDNF-SPIO NP; and the adsorption system of BDNF with and without T80-coating is denoted by T80-BDNF-PBCA-SPIO NP and BDNF-PBCA-SPIO NP. The “protein-plus-cell” approach involves: (1) the administration of neural stem/progenitor cells derived from the nanoparticle-mediated differentiation of iPSCs as a source of cell replacement therapy, and (2) continual treatment with the BDNF-containing nanoparticles to support the reparative process in the CNS, both of which can be tracked by MR imaging.

In this study, a multiplexed PBCA NP delivery platform was constructed to carry BDNF by either surface adsorption or encapsulation; T80-coating was added for CNS targeting, and the magnetic resonance (MR) imaging contrasting agent, superparamagnetic iron oxide (SPIO) was incorporated to facilitate simultaneous non-invasive tracking of the nanoparticles and the treated iPSCs. The synthesized PBCA NPs containing SPIO with surface-adsorbed or encapsulated BDNF, and with or without T80-coating were characterized. The ability of the nanoparticle delivery platform to cross an in vitro BBB-model of rat brain microvascular endothelial cells (RBMECs) and CTX TNA2 (rat astrocyte), and neural differentiation of the exposed iPSCs were assessed. Lastly, the SPIO-tracking capability was tested in a phantom MR experiment. The nanocomposites and the proposed “protein-plus-cell” approach are summarized in Scheme 1.

## 2. Results

### 2.1. Characterization of the Nanoparticles

The particle size and zeta potential measured by the Zetasizer Nano ZS90, and the calculated LEs of SPIO and BDNF for the four types of nanoparticles are shown in Table 1. The effect of surface coating by 0.01% *w*/*v* of T80 on particle size was negligible for both the surface-adsorbed and encapsulated systems of BDNF, while surface adsorption of BDNF on the nanoparticle was responsible for an increased mean diameter from 125 to 150 nm. Modification of the PBCA NPs by T80 and BDNF did not alter the zeta potential, which essentially remained slightly negative to neutral. The fabrication method by acid emulsion polymerization yielded mono-dispersed samples. The preparations that contained T80 were associated with higher SPIO LEs of up to 15%, whereas LEs of greater than 95% were observed for BDNF regardless of its physical location.

**Figure 1:**
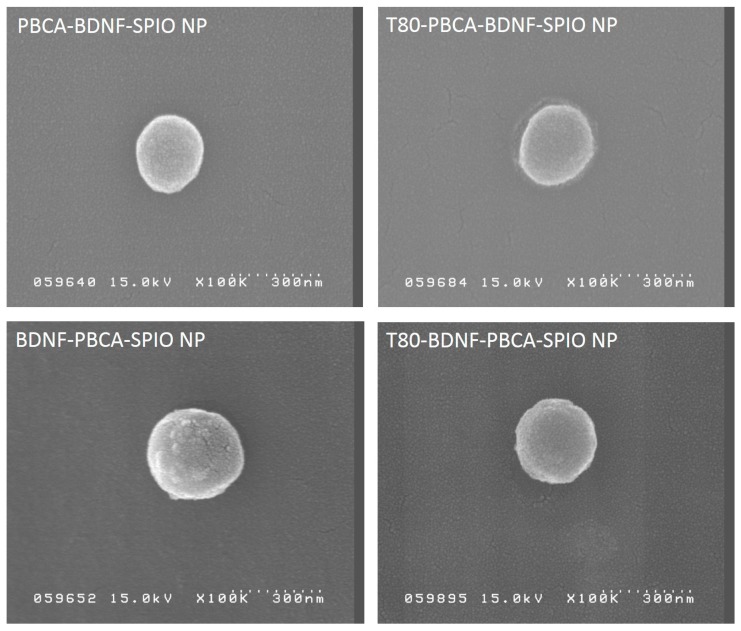
Field emission scanning electron microscope (FE-SEM) images of the nanoparticles at ×100,000 magnification, showing how the nanoparticles are spherical in shape.

**Figure 2:**
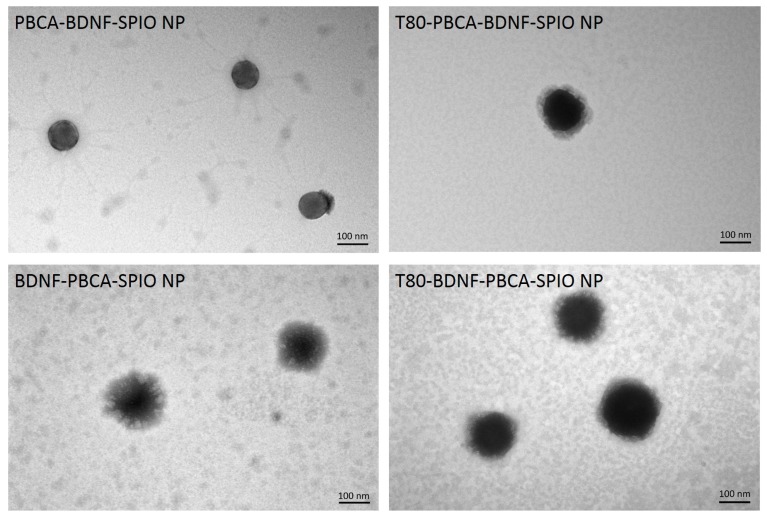
Transmission electron microscope (TEM) images of the nanoparticles at ×200,000 magnification, showing that the nanoparticles have a dense core and an ill-defined halo that is visible for those that contain the surface-adsorbed T80 or BDNF.

The field emission scanning electron microscope (FE-SEM) images showed that the four types of PBCA NPs were spherical in shape (Figure 1), and all had a dense core, as demonstrated in the transmission electron microscope (TEM) images; in addition, and the surface texture was better resolved on the TEM, in which the surface T80 or BDNF conferred an ill-defined halo (Figure 2).

### 2.2. Cytotoxicity of the Nanoparticles on RBMECs, CTX TNA2, and iPSCs

**Figure 3:**
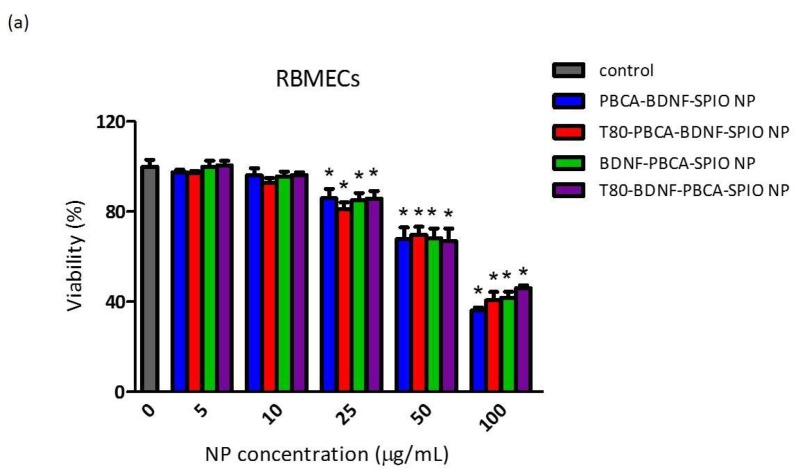

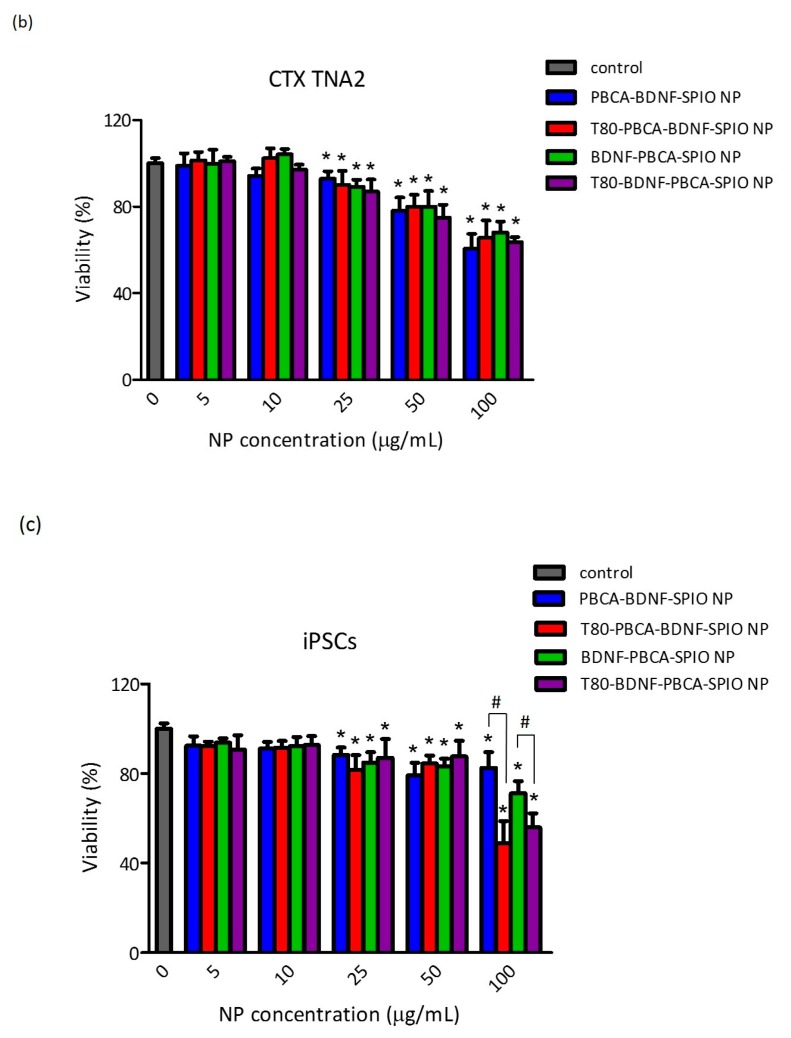
Cell viability determined by the XTT assay for the different PBCA NPs at the dose range of 5 to 100 μg/mL on (**a**) rat brain microvascular endothelial cells (RBMECs), (**b**) rat astrocyte (CTX TNA2), and (**c**) iPSCs. A concentration-dependent cytotoxicity profile is evident, and a concentration of up to 25 μg/mL appears safe for the RBMECs and CTX TNA2, and up to 50 μg/mL for the iPSCs. No significant differences are found in between the adsorption and encapsulation system of BDNF with and without T80 coating across the cell lines, except for the iPSCs in which T80 appears cytotoxic at a high nanoparticle concentration of 100 μg/mL. * *p* < 0.05 to control, ^#^ *p* < 0.001.

The cytotoxicity of the nanoparticles on the RBMECs, CTX TNA2, and iPSCs was determined by the XTT cell viability assay (Figure 3a–c); a concentration-dependent cytotoxicity profile was seen across the different cell types tested, with the RMBECs and CTX TNA2 exhibiting greater than 80% cell viabilities at concentrations of up to 25 μg/mL, and double that for iPSCs. There were no significant differences in cell viabilities amongst the four types of PBCA NPs, except for iPSCs in which cytotoxicity induced by T80 was observed, but only at a high nanoparticle concentration of 100 μg/mL. Therefore, a concentration of 25 μg/mL was chosen for the subsequent cell experiments.

### 2.3. Neural Differentiation of iPSCs Treated with the Nanoparticles

**Figure 4:**
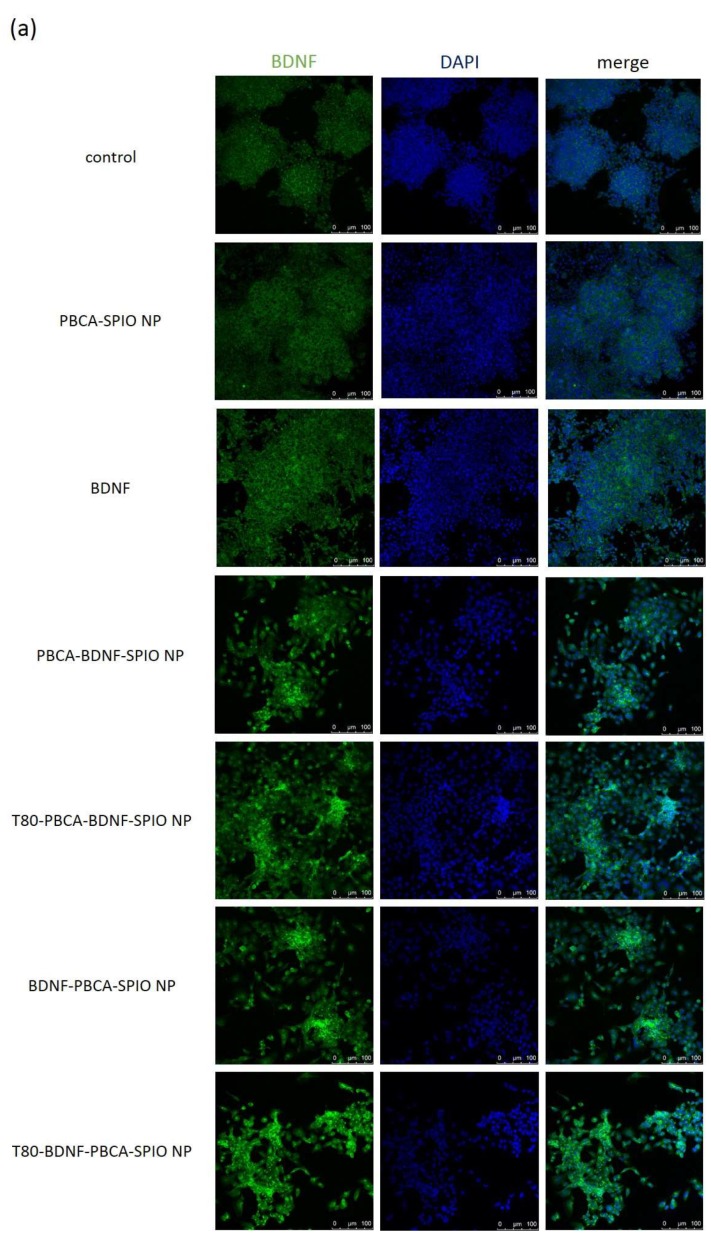

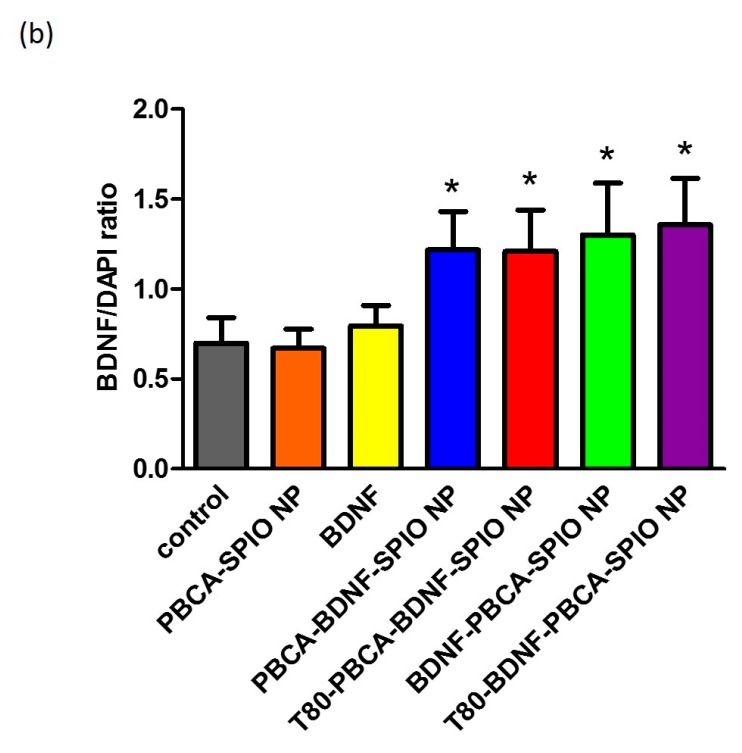
(**a**) Immunofluorescence staining at Day 7 for BDNF (green) of the iPSCs exposed to 125 pg/mL of BDNF alone or 25 μg/mL of nanoparticles with or without BDNF, and (**b**) quantification of BDNF expression by the normalized fluorescence intensity; the levels of BDNF expression appear similar for the control, PBCA-SPIO NPs, and free BDNF, where significant increases can be found for the BDNF-containing nanoparticles, which is associated with a greater dispersion of the cell cluster and elongated cell shape. * *p* < 0.05 to control.

The immunofluorescence staining for BDNF of the iPSCs exposed to BDNF alone or PBCA NPs with or without BDNF is shown in Figure 4a, and quantification by the normalized fluorescence intensity is displayed in Figure 4b; 125 pg/mL of free BDNF was used for the experiment, which contained the equivalent amount of BDNF to 25 μg/mL of PBCA NPs carrying BDNF, assuming 100% loading and release efficiency. Similar degrees of basal BDNF expression were found in the control, PBCA-SPIO NPs, and BDNF alone, whereas treatment with the BDNF-loaded nanoparticles were associated with significant increases in BDNF; however, no appreciable differences amongst the four types of PBCA NPs were found. In addition to the staining intensity of BDNF, cells in those that had the higher level of BDNF appeared more dispersed and elongated in shape.

**Figure 5:**
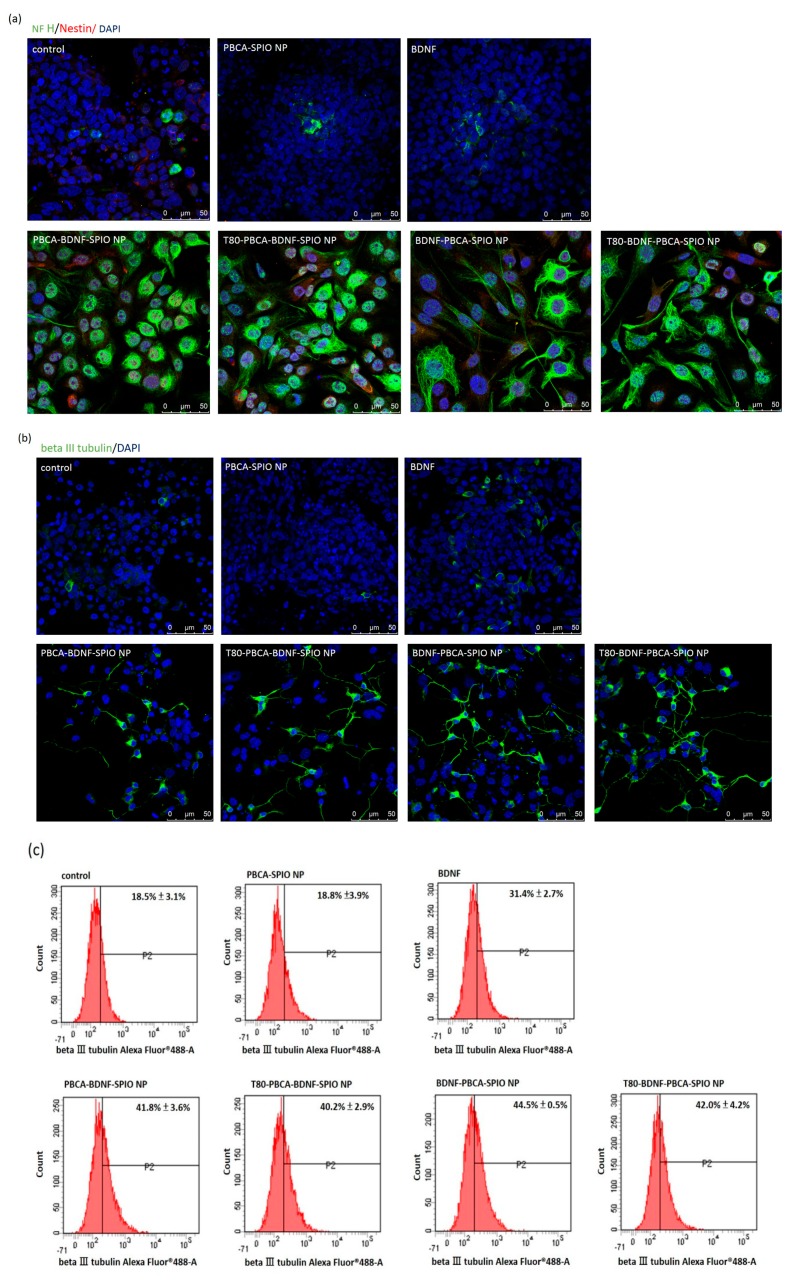
Immunofluorescence staining at Day 7 for (**a**) nestin (red) and the neurofilament-heavy chain (NF H) (green), and (**b**) beta III tubulin (green) on the iPSCs exposed to 125 pg/mL of free BDNF or 25 μg/mL of nanoparticles with or without BDNF. The control, PBCA-SPIO NPs and free BDNF, show a limited number of nestin and NF H-positive cells; by contrast, positively-stained cells are easily identifiable when the BDNF delivery systems are used. Similarly, the control and PBCA-SPIO NP show very few numbers of beta III tubulin-positive cells, and although it is higher with free BDNF, neural morphology cannot be found. Neural differentiation is clearly seen in cells treated with the BDNF-containing nanoparticles; furthermore, surface-adsorbed BDNF is associated with a greater degree of axon elongation and intercellular connections. (**c**) Flow cytometry analysis of beta III tubulin-positive cells on iPSCs exposed to free BDNF or nanoparticles; the control and PBCA-SPIO NPs show similar percentages. This percentage increases to 31.4 ± 2.7% with free BDNF, and further increases to 41.9 ± 3.4% with the BDNF delivery systems.

Neural differentiation of the iPSCs was assessed by immunofluorescence staining at Day 7 against the neural stem/progenitor cell markers, nestin and the neurofilament-heavy chain (NF H), as well as the early neural differentiation marker beta III tubulin, following exposure to free BDNF or nanoparticles with or without the loaded BDNF. Images captured by confocal microscopy are displayed in Figure 5a,b; cells that express nestin and NF H were scant in the control as well as those treated with free BDNF and PBCA-SPIO NPs, whereas numerous positively stained cells were seen when the BDNF-loaded nanoparticles were given. Likewise, the control and those treated with PBCA-SPIO NPs contained very few cells positive for beta III tubulin, and only slightly more were found with BDNF alone; moreover, cells of neural morphology could not be identified—by contrast, neural differentiation of cells treated with the BDNF-containing nanoparticles was clearly distinguishable, as these cells were not only stained strongly for beta III tubulin but also displayed a multipolar morphology, consisting of a single axon and multiple dendrites projecting from the cell body. Furthermore, a better neural differentiation was seen in cells treated with the nanoparticles that had the surface-adsorbed BDNF than the encapsulated formulation, which was associated with a greater degree of axon elongation and intercellular connections. The presence or absence of T80 coating did not appear to qualitatively influence the neuron morphology.

The flow cytometric detection of beta III tubulin-positive cells derived from the iPSCs treated with the respective nanoparticles and BDNF alone is shown in Figure 5c; the percentage of beta III tubulin-positive cells with PBCA-SPIO NPs was 18.8 ± 3.9%, which was similar to the control (*p* = 0.92), this percentage rose to 31.4 ± 3.1% for BDNF only (*p* < 0.001), and 41.9 ± 3.4% for the BDNF-containing nanoparticles, which equated to a 1.3-fold increase when compared to the control (*p* < 0.001); however, there were no significant differences amongst the different delivery systems (*p* = 0.303).

### 2.4. The Effect on the Transendothelial Electrical Resistance and Permeability of the Nanoparticles across the In Vitro Blood–Brain Barrier Model

**Figure 6:**
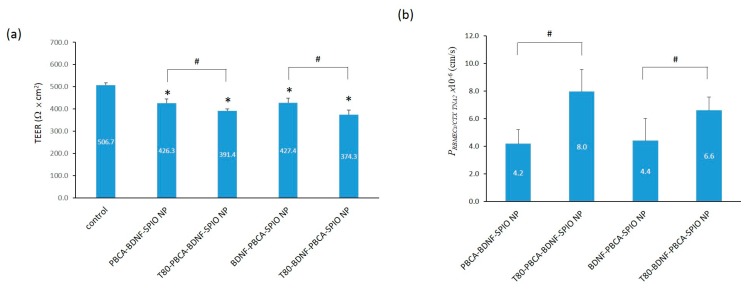
(**a**) The transendothelial electrical resistance (TEER) (Ωcm^2^) across the contact co-culture system of RBMECs and CTX TNA2 for the BDNF-containing PBCA-SPIO NPs at 25 μg/mL for 4 h. The TEER of the treated cells are significantly lower than the control, and the values of the adsorption and the encapsulation system of BDNF only differ with respect to the presence and absence of T80 coating, which, on average, measures 382.9 ± 18.1 and 426.9 ± 19.7 Ωcm^2^, respectively. (**b**) The permeabilities of the nanoparticles (cm/s) correspond to the degree of reduction in the TEERs, which are significantly higher with T80 coating regardless of the physical location of BDNF. * *p* < 0.05 to control, ^#^ *p* < 0.05.

The measured transendothelial electrical resistance (TEER) of the stabilized in vitro BBB modeled by the transwell system of contact co-cultured RBMECs and CTX TNA2 was 506.7 ± 11.5 Ωcm^2^. Significant reductions in TEERs from baseline signifying disruptions on the integrity of the BBB were demonstrated when the nanoparticles were added to the RBMEC side of the system. The TEERs of the systems exposed to nanoparticles that contained surface-adsorbed or encapsulated BDNF without T80 did not differ significantly, which averaged to 426.9 ± 19.7 Ωcm^2^, but an 8% reduction to 382.9 ± 18.1 Ωcm^2^ was observed with T80-coated nanoparticles (*p* < 0.05) (Figure 6a).

The permeability of the nanoparticles across the in vitro BBB model was represented by the permeability coefficient, which was quantified by the fluorescence intensity of the fluorescein isothiocyanate (FITC)-labeled nanoparticles collected on the side of the CTX TNA2 (Figure 6b). An inverse relationship of the permeability to the TEER was demonstrated; again, the permeabilities of the BDNF-containing nanoparticles only differed by the presence or absence of T80 (7.3 ± 1.5 × 10^−6^ versus 4.3 ± 1.3 × 10^−6^ cm/s, *p* < 0.001) and were not affected by the physical location of the BDNF; coating with the T80 lead to a 1.5- to 2-fold increase in permeability from baseline, and a 70% increase when compared with the uncoated formulation.

### 2.5. Prussian Blue Staining for Detection of SPIOs

**Figure 7:**
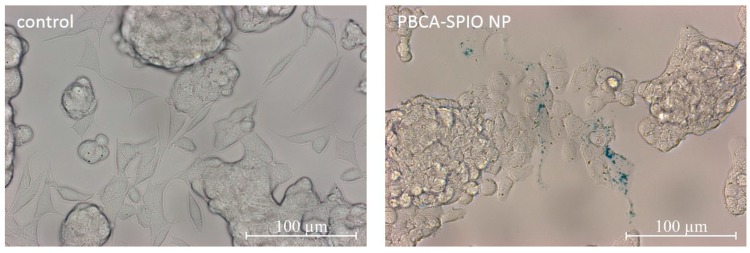
Prussian blue stain of iPSCs treated with 25 μg/mL of the PBCA-SPIO NP under 400× magnification shows cellular internalization of the SPIOs, which is visualized as small solid blue aggregates within the cell cytoplasm.

Prussian blue staining for the detection of iron inside the iPSCs treated with the PBCA-SPIO NPs for 4 h is shown in Figure 7; the staining technique identified cells containing SPIOs by the precipitation of ferric iron from the SPIOs with the soluble ferrocyanide in the stain, which were characterized by the presence of solid blue deposits within the cytoplasm.

### 2.6. In Vitro MR Relaxitivities of the Nanoparticles

**Figure 8:**
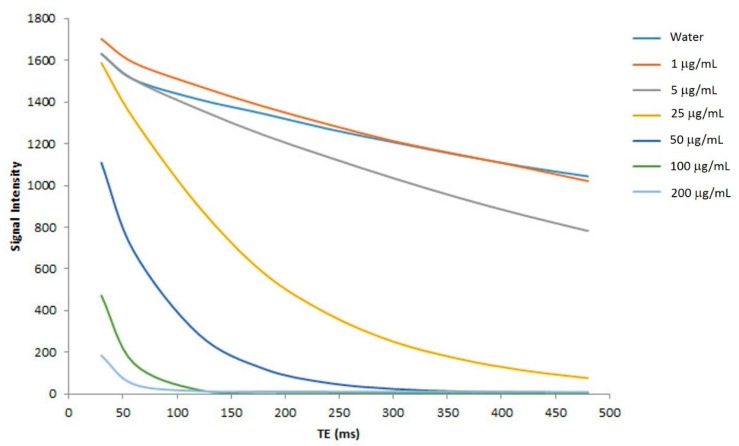
The phantom MR measurements of different concentrations (1 to 200 μg/mL) of PBCA-SPIO NPs are represented on the signal intensity versus TE plot. The signal intensity decreases with increasing iron content (PBCA-SPIO NP concentration), and the rate of decay also becomes faster.

Phantom MR experiments were applied to investigate the magnetic property of PBCA-SPIO NPs. The MR signal intensity versus echo time (TE) at various concentrations of PBCA-SPIO NPs was plotted (Figure 8). The signal intensity and relaxitivity at a low concentration of 1 μg/mL was not too dissimilar from water; however, the increase in nanoparticle concentration led to a progressive decline in signal intensity, as well as a faster rate of decay.

## 3. Discussion

A polybutylcyanoacrylate nanoparticle is a biocompatible, biodegradable synthetic polymeric drug delivery system [39,40]. It is the first polymer-based nanoparticle system tested to deliver drugs into the CNS [41]. The main building block of the PBCA NP is the BCA monomer, which polymerizes and solidifies when placed in contact with anions or radicals present on tissue surfaces or fluids [42]; thus, it is clinically approved as a topical adhesive for skin closure, as a surgical hemostatic agent, and liquid embolic agent for endovascular intervention [43,44,45]. PBCA NP degrades in the biological system by esterase-mediated hydrolysis of the ester side chains, which is a pH-dependent process with half-lives that range from 144 days at pH 4 to 3 days at pH 7.4; therefore, it can resist breakdown in the acidic environment of the lysosome and effectively deliver its payload into the cytosol [46,47]. PBCA NP can be modified according to clinical need; for example, PEGylation of PBCA NP evades clearance by the reticuloendothelial system, which enables increased circulation time for passive targeting and potentiates the therapeutic effect at lower doses [48]; surface coating with T80 alters the bio-distribution preferentially for the brain [49], and simultaneous loading of different substances by surface adsorption and encapsulation permits multiplexing capabilities. The loading of substances on and within the PBCA NPs is achieved sequentially; first by an incorporation step that encapsulates the desired content in the reagent mixture into the nanoparticles during the acid emulsion polymerization process, and a second incubation step by which the substance to be carried by surface adsorption is added to the solution of formed nanoparticles. These synthetic steps enable the optimal loading efficiency of BDNF for both the adsorption and the encapsulation systems; however, the use of the magnetic stirrer, coupled with a low stirring speed during the incorporation step, could reduce the available SPIO in the homogenate and lead to a low LE. Conversely, the inclusion of T80 could produce a better stabilized system of nanoparticles, and allow for the additional loading of SPIO by surface adsorption during the incubation step, thus resulting in an increased LE.

The BBB has an important regulatory function on the microenvironment of the CNS, but it also restricts entry of therapeutic substances from the circulation [50,51]. Various in vitro cell models can be used to simulate the BBB, the basic component of which comprises of the neurovascular unit made of brain capillary endothelial cells surrounded by a basement membrane, astrocytes, and pericytes [52]. The BDNF-containing nanoparticles were screened for toxicity on the cell lines that constitute the in vitro BBB model as well as on the iPSCs; a concentration of 25 μg/mL was considered safe for all the cell lines regardless of the presence or absence of T80 coating, and surface-adsorbed or encapsulated BDNF; notably, the iPSCs were capable of withstanding a dose of up to 100 μg/mL, but showed additional T80 toxicity at this dose in spite of the low concentration of T80 (0.01% *w*/*v*) used, which was considerably less than the usual range of 0.1% to 10% (*w*/*v*) [53,54,55,56]. Although T80 is a commonly used surfactant, a previous study indicates a lethal concentration 50% (LC_50_) of 0.021% (*w*/*v*) on human fibroblasts; therefore, the toxicity of T80 should not be underestimated [57]. The integrity of the BBB can be assessed by the TEER, which is a measure of the electrical resistance across the cell layer through the transcellular and paracellular pathways in parallel; the resultant TEER of 506.7 ± 11.5 Ωcm^2^ in our contact co-culture system of RBMECs and CTX TNA2 is consistent with those reported in the literature [58,59], and although higher TEER in excess of 2000 Ωcm^2^ like those found in vivo can be achieved by increasing the complexity of the modeled system, these generally reflect on the integrity of the tight junctions; thus, values of 160 to 200 Ωcm^2^ or greater are generally considered adequate for BBB screening, which may be sufficient for testing nanoparticles that are too large to traverse the paracellular pathway in the presence of intact tight junctions [60,61,62]. The T80-coated and bare PBCA NPs have been shown to cause a transient reduction in the TEER by 80% and 50% from the baseline, respectively, and it is completely recoverable in the former but only partially in the latter. This change is attributed to an alteration in cell morphology and the organization of the neurovascular unit induced by the nanoparticles, leading to an opening of the paracellular pathway [63,64]. We were able to show that even at a much lower concentration of T80 (0.01% *w*/*v*), the T80-coated formulations can still cause a small but significant decline in the TEER than those without T80 (24% versus 16%, *p* < 0.05). The corresponding increase in permeability of the nanoparticles across the cell layer in the transwell system was reflected proportionately to the reduction in TEER, and the inclusion of surface-adsorbed BDNF did not appear to interfere with the transport process. The permeability coefficients of the nanoparticles were within range of the typical transwell assay, which are in the order of 10^−7^ to 10^−3^ cm/s, and are beyond the threshold of 1.5 to 2.0 × 10^−7^ cm/s recommended for CNS therapeutics [65,66]. Transport of PBCA NPs across the BBB may take place via the transcellular pathway, which is believed to be the main route of transport; however, the subsequent loosening of tight junctions may also accommodate for transport through the paracellular pathway. The transcellular route is mediated by ligand-receptor interaction; the surface of these T80-coated nanoparticles may act as anchor points for apolipoproteins, and these apolipoprotein-nanoparticle complexes are then taken up by brain capillary endothelial cells through the interaction with lipoprotein receptor or lipoprotein receptor-related proteins on the luminal surface of the plasma membrane [32,67]. Alternatively, T80 may boost the fixed charge density on the surface of nanoparticles and increase their electrophoretic mobility and zeta potential. This, together with a surfactant layer on the nanoparticles, shifts the slip plane towards the solid surface, meaning that the attraction energy between the nanoparticle and the cell necessary for adsorption-mediated uptake is enhanced [68]. The slightly negatively-charged to neutral zeta potential of the nanoparticles in this study may still be capable of cell penetration, which can outperform positively-charged species due to strong and non-specific interactions with the plasma membrane [69].

The contrast in an MR image is a function of the different signal intensities from the volume elements; the signal intensity is dependent on the water proton density and the longitudinal (T_1_) and transverse (T_2_) relaxation times of the excited proton spins to the ground state; the relaxation times of protons in the target tissue are influenced by the presence of MR contrast agents that can be broadly classified as T_1_ or T_2_-contrast agents according its predominant effect on the relaxation times. SPIO is a type of T_2_-constrast agent that causes marked reductions in T_2_-relaxation time and signal intensity, and the results in our dose escalation phantom MR experiments of PBCA-SPIO NPs were comparable with those reported in the literature [70]. The nano-system-based iron oxide not only improves its dispersity and colloidal stability, but also increases the net magnetic moment to optimize the saturation magnetization, such that the T_2_-relaxitivity can be shortened for a greater MR contrast [71,72]. The inclusion of SPIO in our delivery system can potentially be used to study the fate of the nanoparticles or the nanoparticle-treated iPSCs by MR imaging; furthermore, the presence of SPIO in the nanoparticles could offer the option of targeted delivery by using an externally applied magnetic field across the site of interest [73].

Various in vitro differentiation protocols have been described for the derivation of neurons from iPSCs, which often require multiple inductive agents in a multi-step process that can take weeks to months [74,75,76]. The lengthy step in obtaining iPSCs by the reprogramming of somatic cells, followed by the protracted differentiation process associated with the possible need for embryoid body formation under undefined stromal co-culture systems, and the low yield of neurons with variable maturation stages and functional properties, limits the clinical utility of autogenic iPSC-derived cell products as a source of cell replacement therapy [27,77,78]. Dual inhibition of the SMAD pathway can be utilized to overcome these problems [79,80], which relies on the synergistic action of Noggin, a bone morphogenetic inhibitor, and small molecule SB431542, an anaplastic lymphoma kinase inhibitor to block phosphorylation of SMAD-1, 5, 8 and SMAD-2, 3 of the respective pathways, and ultimately affect the downstream formation of SMAD complex in a way that the pattern of gene expression is driven towards neural differentiation [81]; greater than 80% differentiation efficiency by dual SMAD inhibition can be achieved within 19 days under adherent culture conditions [82]. Alternatively, the one-step forced expression of the master neural transcriptional regulator neurogenin-2 by lentiviral transduction of iPSCs has been reported to produce a homogenous population of mature neurons within 2 to 3 weeks, with nearly 100% differentiation efficiency of the transduced cells [83,84,85]. The envisioned “protein-plus cell” approach seeks to shorten the preparatory time window, and aims for the transplantation of cells at the earliest possible differentiation stage, followed by continual neutrophin stimulus through the ongoing administration of BDNF-containing nanoparticles to further assist in healing. Neurotrophins such as BDNF are the key regulators of cell fate and function in the nervous system [86]. We previously reported the forced expression of BDNF in iPSCs by the non-viral transduction of BDNF gene using a PBCA NP delivery system which resulted in neuron-like cells in as early as 7 days, and showed that activation of the phosphatidylinositol-3-OH kinase/Akt kinase pathway is capable of neural lineage specification [87]. In this study, BDNF-containing PBCA NPs were developed as an alternative to BDNF gene transduction; the use of these nanoparticles resulted in distinct cell populations distinguishable by immunofluorescence for neural markers and morphology in as early as 7 days consisting of large irregularly shaped neural stem/progenitor cells, small neuron-like cells, and compact rounded cells resembling undifferentiated iPSCs, as would be expected during the early days of the differentiation process. This was in agreement with the flow cytometric analysis, showing only slightly greater than 40% of cells bearing beta III tubulin. Although the treated cells showed evidence of neural differentiation, the morphologic differences, with respect to axonal and dendritic growth and intercellular connections, were much more extensive in those treated with surface-adsorbed BDNF. This is consistent with the fact that BDNF acts on the extracellular domain of the tropmycin kinase B receptor located in the cell membrane [88]; therefore, the ease of BDNF release into the extracellular compartment before the nanoparticles are taken up for cell tracking will optimize the ligand-receptor interaction to activate downstream pathways. Treatment with an equivalent concentration of free BDNF did not result in appreciable neural differentiation; this was likely caused by the rapid degradation of BDNF in vitro without the protection of the delivery system [54]. The elevated levels of BDNF expression seen in iPSCs treated with the BDNF-containing nanoparticles has been described as a self-amplifying, positive-feedback autocrine mechanism during neural development to ensure an adequate level of BDNF activity necessary for neural differentiation and growth [89]. The one-time administration and limited half-life of free BDNF in vitro may explain the lack of findings for the cells treated with BDNF alone.

Even though the adsorption system worked well in vitro, it may be less suitable for delivery in vivo owing to the expected burst release profile and the short half-life of BDNF in plasma; thus, the encapsulation system may have the advantage of preventing losses during its transit to the target [90].

We have demonstrated a multiplexed PBCA NP delivery system for BDNF and SPIO with the potential for simultaneous induction of neural differentiation and tracking capability of the treated iPSCs. T80 coating, even at minute concentrations of 0.01% (*w*/*v*) allowed for increased permeability of the delivery system across the in vitro contact co-culture BBB model made up of RBMECs and CTX TNA2. Neural differentiation appeared best with those containing the adsorbed BDNF, consistent with its site of interaction at the extracellular domain of the tropmycin kinase B receptor, and the derivation process may be as short as 7 days. However, the 40% differentiation efficiency by the one-time administration of BDNF-containing PBCA NPs over free BDNF on the neural differentiation of mouse iPSCs is far from perfect, and many questions and challenges lie ahead before the “protein-plus-cell” approach can be realized; these include the possible differences in inductiveness on different clones of iPSCs, and, more importantly, on things such as human iPSCs, the fate of the treated iPSCs on a longer time scale, the effect of repeated exposure to the BDNF-bearing nanoparticles, the requirement of additional extrinsic factors other than BDNF for functional maturation and derivation of neural subtypes, better characterization of the treated cells by methods such as transcriptome analysis, and ultimately, the relevance of such findings to in vivo disease models.

## 4. Materials and Methods

### 4.1. Mouse iPSCs

Mouse iPSCs were purchased from System Biosciences (Mountain View, CA, USA). The iPSCs were maintained in ESGRO complete PLUS clonal grade medium (Millipore, Billerica, MA, USA). When the culture reached 80–90% confluence, the cells were passaged at a ratio of 1:4 in 75 cm^2^ tissue culture flasks pre-coated with 0.1% gelatin. Cells of passages 14–18 were used for all the experiments.

### 4.2. Synthesis of SPIO

For the synthesis of SPIO particles, 1.83 g FeCl_3_·6H_2_O and FeCl_2_·6H_2_O were dissolved in 25 mL of deionized water. The solution was stirred rigorously for 5 min at room temperature after the slow addition of 4 mL 25% ammonia aqueous solution. The formation of iron oxide was indicated by the solution’s change in color from brown to black. The precipitant was isolated by a permanent magnet, which was then re-dispersed and washed with deionized water. This procedure was repeated three times to purify the synthesized iron oxide particles. Finally, the SPIO was stored in 40 mL of deionized water for further experimentation.

### 4.3. Fabrication of PBCA-BDNF-SPIO NP (BDNF Encapsulation System) and BDNF-PBCA-SPIO NP (BDNF Adsorption System) with and without T80 Coating

PBCA NPs entrapping SPIO and BDNF were prepared by the emulsion polymerization technique; the fabrication method was reported previously with minor modifications [53,91]. Briefly, the PBCA NPs entrapping SPIO were made by adding 1% (*v*/*v*) BCA (Sicomet, Sichel Werk, Hanover, Germany) into a 0.01 N HCl acidic polymerization medium containing 1% (*w*/*v*) dextran 70,000 (Fluka Biochemika, Buchs, Switzerland) and 0.002% (*w*/*v*) SPIO; the solution was kept at 25 °C and stirred at 400 rpm for 3 h. Polymerization was terminated by neutralization with 0.1 N NaOH; the suspension was filtrated through a 1 μm filter paper to remove the larger polymer aggregates. 200 ng/mL BDNF (R&D Systems, Minneapolis, MN, USA) was included in the initial polymerization medium for the encapsulating system of BDNF, whereas the same amount of BDNF was added to the suspension of formed PBCA-SPIO NPs and stirred for 1 h at 400 rpm for the adsorption system of BDNF. For the T80-coated formulation of the respective systems, the nanoparticles were further incubated in 0.01% (*w*/*v*) of T80 and stirred for another 30 min. The excess unloaded agents in each preparation were removed by ultrafiltration (Amicon Ultra-4 30K devices, Millipore, Billerica, MA, USA).

FITC labeling of the nanoparticles was achieved by replacing dextran 70000 with FITC-dextran (Fluka Biochemika, Buchs, Switzerland) in the reaction system. Sterilization of the nanoparticle formulation was achieved by filtering through a 0.22 μm filtration unit (Millipore).

### 4.4. Characterization of Nanoparticles

The particle size and zeta potential of the nanoparticles were determined by the Zetasizer Nano ZS90 (Malvern, Worcestershire, UK) with photo correlation spectroscopy. The nanoparticle morphology was obtained by a FE-SEM (S-5000, Hitachi, Tokyo, Japan), and by a TEM (H-7500, Hitachi) after negative staining with 2% (*w*/*v*) phosphotungstic acid (Sigma, St. Louis, MO, USA) for a strong contrast in the images.

### 4.5. Loading Efficiency of BDNF and SPIO

The free BDNF in solution was analyzed with a human BDNF enzyme linked immunosorbent assay (ELISA) kit (R&D Systems) after ultrafiltration. The absorbance was measured by a spectrophotometer (ELISA reader) set to a wavelength of 450 nm. The LE of BDNF by the nanoparticles is given by the formula: *LE*_BDNF_ (%) = (total weight of BDNF − weight of BDNF in supernatant)/total weight of BDNF × 100.

The LE of SPIO was obtained by thermogravimetric analysis using a Mettler TGA/SDTA 851 (Mettler Toledo, Columbus, OH, USA) after freeze-drying.

### 4.6. Cytotoxicity Assay

The cytotoxicity of the nanoparticles to RBMECs, CTX TNA2, and iPSCs was estimated by the XTT cell viability assay (Cell Proliferation kit, Biological Industries, Beit Haemek, Israel). Cells were seeded onto a 96-well plate at a density of 5000 cells/well and incubated overnight. The cells were then treated with different concentrations of nanoparticles in 100 µL growth medium and incubated for 2 h, after which the tetrazolium dye for the XTT assay was added and incubated for 24 h. The reduced derivative of the tetrazolium dye was read by the EnSpire Multimode Plate Reader (Perkin Elmer Inc., Waltham, MA, USA) with the absorbance wavelength set at 490 nm.

### 4.7. Immunofluorescence Staining and Flow Cytometry Analysis

The iPSCs were seeded at a density of 1 × 10^5^ cells/cm^2^ on a 0.1% gelatin-coated culture dish and cultured with ESGRO complete PLUS clonal grade medium in a humidified 5% CO_2_ incubator at 37 °C overnight. The iPSCs were cultured with 125 pg/mL of free BDNF or 25 μg/mL of PBCA-SPIO NPs with and without BDNF for 7 days; the concentration of the free BDNF was taken as the equivalent of 25 μg/mL of BDNF-containing nanoparticles, assuming 100% loading and release efficiency. The cells were fixed with 4% formaldehyde for 10 min and stained with the primary antibody against BDNF (1:1000, Abcam, Cambridge, MA, USA), beta III tubulin (1:200, Abcam), NF H (1:200, Abcam), and Nestin (1:200, Abcam) at 4 °C overnight, and detected with an Alexa Fluor^®^ 488 (1:1000, Abcam)-conjugated or Alexa Fluor^®^ 594 (1:1000, Abcam)-conjugated secondary antibody at room temperature for 1 h. The nuclei of iPSCs were counterstained with 4′, 6-diamidino-2-phenylindole (DAPI). The images were captured by a confocal laser-scanning microscope (Leica TCS SP5 II, Wetzlar, Germany). The fluorescence intensity of BDNF was quantified by imageJ analysis, normalized to the fluorescence intensity of DAPI.

Flow cytometry was used to calculate the percentage of iPSCs differentiating into neurons for each of the respective treatment groups at Day 7. The cells were fixed with 4% formaldehyde for 10 min and treated with 0.1% (*v*/*v*) Triton X-100 in DPBS for 10 min. After washing with DPBS, the cells were reacted with rabbit polyclonal anti-beta III tubulin (1:100, Abcam) at 4 °C for 1 h, and goat polyclonal secondary antibody to rabbit IgG-H&L Alexa Flour^®^ 488 conjugate (1:4000, Abcam) at 4 °C for 30 min. Then, the fluorescence-labelled cells were stabilized by suspension in 200 L DPBS with 1% (*v*/*v*) formaldehyde at 4 °C. Processed single-cell suspensions were detected by a BD FACSCanto™ II flow cytometer (BD Biosciences, California, CA, USA) and analyzed by Cell Quest software (BD Biosciences).

#### 4.7.1. In Vitro BBB Model by Co-Culturing Endothelial Cells (Upper Chamber) and Astrocytes (Opposite the Upper Chamber)

The isolation of RBMECs and the cultivation of RBMECs and CTX TNA2 were described previously [92]. The top and bottom surfaces of a sterilized polyethylene terephthalate (PET) membrane (cell culture insert, pore size 1.0 μm, BD, Franklin Lakes, NJ, USA) were coated with human fibronectin (Sigma), and 1 × 10^5^ cells/cm^2^ of CTX TNA2 (Bioresource Collection and Research Center; no. 60547, Hsin-Chu, Taiwan) were seeded on the bottom surface of the PET membrane in an inverted transwell system, and cultivated in the CO_2_ incubator for 30 min; then, 1 × 10^5^ cells/cm^2^ of RBMECs were seeded on the top surface of the PET membrane. The resultant transwell system of RBMECs/CTX TNA2 was placed in a 24-well micro-titer plate (BD Biosciences) and co-cultivated in the CO_2_ incubator over 10 days to form a confluent monolayer of RBMECs regulated by CTX TNA2. The lower (outer) chamber was filled with the Dulbecco’s Modified Eagle Medium (DMEM) (Gibco, Grand Island, NY, USA) with 10% fetal bovine serum (FBS) (Hyclone, Logan, UT, USA) for CTX TNA2. The medium in the upper (inner) chamber was the endothelial cell medium (ECM, ScienCell, Corte Del Cedro Carlsbad, CA, USA) containing 1% endothelial cell growth supplement, 10% FBS, and 1% penicillin/streptomycin solution. Both media were replaced every 2 days and maintained at the same height to reduce the effect of hydrostatic pressure on RBMECs and CTX TNA2.

#### 4.7.2. TEER of RBMECs/CTX TNA2 Monolayer after Treatment with the Nanoparticles

The donor and receiver chambers of the transwell system faced the RBMECs and CTX TNA2, respectively. A nanoparticle concentration of 25 μg/mL in the ECM was added to the donor chamber and cultured in the humidified CO_2_ incubator at 37 °C for 4 h. The donor and receiver chambers were filled with the respective culture media to the same height. A Millicell voltohmmeter (Millipore) was applied to determine the TEER of the PET insert with and without the RBMECs/CTX TNA2.

#### 4.7.3. Permeability Across Co-Cultured RBMECs/CTX TNA2 System

The transwell system containing the RBMECs/CTX TNA2 was used to determine the permeability coefficient of FITC-labeled nanoparticles across the CTX TNA2-regulated monolayer of RBMECs (*P*_RBMECs/CTX TNA2_). The donor chamber was given 25 μg/mL of nanoparticles and placed in the humidified CO_2_ incubator. After 4 h, the concentration of FITC-labeled nanoparticles in the receiver chamber was analyzed by the EnSpire Multimode Plate Reader, set at the emission and excitation wavelengths of 525 nm and 488 nm, respectively. The medium in the receiver chamber was compensated with fresh DPBS when sampling. The *P*_RBMECs/CTX TNA2_ was calculated by 1/*P*_RBMECs/CTX TNA2_ = 1/*P*_e_ − 1/*P*_m_, where *P*_e_ and *P*_m_ represent the permeability of FITC-labeled nanoparticles across the PET membrane containing RBMECs/CTX TNA2 and the permeability of the same nanoparticles across the cell-free PET membrane, respectively.

### 4.8. Prussian Blue Staining for Detection of SPIOs

The iPSCs were seeded at a density of 1 × 10^5^ cells/cm^2^ on a 0.1% gelatin-coated culture dish and cultured with ESGRO complete PLUS clonal grade medium in a humidified 5% CO_2_ incubator at 37 °C overnight. The iPSCs was treated with 25 μg/mL PBCA-SPIO NPs for 4 h, then fixed with 4% formaldehyde and washed with 1× PBS; these were further incubated with a 1:1 mixture of 4% potassium ferrocyanide and 4% hydrochloride acid for 30 min. The cells were observed under a light microscope (Zeiss Axio Observer, Oberkochen, Germany).

### 4.9. In Vitro MRI Signal Intensity of the Nanoparticles

MR tests using PBCA-SPIO NPs were performed to assess the efficacy of the probe as a T2-weighted MR imaging contrast agent. PBCA-SPIO NPs of various concentrations (1, 5, 25, 50, 100, and 200 μg/mL) were suspended in 1.5 mL eppendorfs containing phosphate buffer saline solution. The tubes were embedded in a homemade tank filled with 1% agarose gel. The tank with one eppendorf was inserted into the MR coil with the long axis of the vials parallel to the static magnetic field. T2-weighted MR images were acquired on a Burker 7.0-T small animal MR imaging system (ClinScan, Bruker, Billerica, MA, USA) with a fast spin echo sequence under the following parameters: TR = 4000 ms; TE = 109 ms with 16 echo-train length.

### 4.10. Statistical Analysis

The numeric variables were expressed as mean ± standard deviation. All statistical analyses were performed with the GraphPad Prism^®^ 5 software (La Jolla, CA, USA). The data were analyzed with the one-way analysis of variance (ANOVA) and Bonferroni post-hoc test. A *p* value of 0.05 or less was thought to indicate a significant statistical difference.

## 5. Conclusions

Neural differentiation from iPSCs can be induced by the administration of BDNF carried by PBCA NPs, and the multiplexing capabilities of this polymeric delivery platform allows for further modification, such as the inclusion of SPIO for added MR tracking functionality and T80 coating for enhanced penetration through the BBB. Treatment of iPSCs with surface-adsorbed BDNF nanoparticles was found to give rise to cells with better axonal and dendrite growth and intercellular connectivity, which was noticeable in as early as 7 days, compared to the encapsulated formulation.

## Figures and Tables

**Table 1:** The average diameter (Dav), zeta potential, polydispersity index (PDI), and loading efficiencies of BDNF and SPIO for the four types of nanoparticles.

Sample	Dav (nm)	Zeta Potential (mV)	PDI	Loading Efficiency (%)	
				SPIO	BDNF
PBCA-BDNF-SPIO NP	125.2 ± 3.2	−1.3 ± 0.3	0.17 ± 0.01	10.96	100
T80-PBCA-BDNF-SPIO NP	124.5 ± 0.8	−0.2 ± 1.0	0.13 ± 0.04	15.32	100
BDNF-PBCA-SPIO NP	148.7 ± 0.4	−0.4 ± 0.2	0.12 ± 0.02	7.78	98.29 ± 0.16
T80-BDNF-PBCA-SPIO NP	146.7 ± 1.1	−1.4 ± 0.1	0.13 ± 0.02	14.16	98.96 ± 0.33
